# The adoption and use of learning analytics tools to improve decision making in higher learning institutions: An extension of technology acceptance model

**DOI:** 10.1016/j.heliyon.2024.e26315

**Published:** 2024-02-18

**Authors:** Muaadh Mukred, Umi Asma’ Mokhtar, Burkan Hawash, Hussain AlSalman, Muhammad Zohaib

**Affiliations:** a Department of Business Analytics, Sunway Business School, Sunway University, 5, Jalan University, Bandar Sunway, 47500, Petaling Jaya, Selangor, Malaysia; b Faculty of Information Science and Technology, Universiti Kebangsaan Malaysia, 43600 Bangi, Selangor, Malaysia; c Department of Computer Science, College of Computer and Information Sciences, King Saud University, Riyadh 11543, Saudi Arabia; d Software Engineering Department, Lappeenranta-Lahti University of Technology, 53851 Lappeenranta, Finland

**Keywords:** Learning analytics, Higher learning institutions, Technology adoption, Technology acceptance model, Decision-making

## Abstract

Learning Analytics Tools (LATs) can be used for informed decision-making regarding teaching strategies and their continuous enhancement. Therefore, LATs must be adopted in higher learning institutions, but several factors hinder its implementation, primarily due to the lack of an implementation model. Therefore, in this study, the focus is directed towards examining LATs adoption in Higher Learning Institutions (HLIs), with emphasis on the determinants of the adoption process. The study mainly aims to design a model of LAT adoption and use it in the above context to improve the institutions' decision-making and accordingly, the study adopted an extended version of Technology Acceptance Model (TAM) as the underpinning theory. Five experts validated the employed survey instrument, and 500 questionnaire copies were distributed through e-mails, from which 275 copies were retrieved from Saudi employees working at public HLIs. Data gathered was exposed to Partial Least Square-Structural Equation Modeling (PLS-SEM) for analysis and to test the proposed conceptual model. Based on the findings, the perceived usefulness of LAT plays a significant role as a determinant of its adoption. Other variables include top management support, financial support, and the government's role in LATs acceptance and adoption among HLIs. The findings also supported the contribution of LAT adoption and acceptance towards making informed decisions and highlighted the need for big data facility and cloud computing ability towards LATs usefulness. The findings have significant implications towards LATs implementation success among HLIs, providing clear insights into the factors that can enhance its adoption and acceptance. They also lay the basis for future studies in the area to validate further the effect of LATs on decision-making among HLIs institutions. Furthermore, the obtained findings are expected to serve as practical implications for policy makers and educational leaders in their objective to implement LAT using a multi-layered method that considers other aspects in addition to the perceptions of the individual user.

## 1 Introduction

In the modern education system, Learning Analytics has found its niche in enhancing decision-making by providing insights using student learning and engagement data. More specifically, Learning Analytics Tools (LATs) are described as distinct applications and software programs utilized for collecting, analyzing, and reporting data for learning. They are educational tools that are useful for students, teachers, and the overall learning institution [[1], [2], [3], [4], [5], [6]].

Moreover, due to its ability to track students' learning outcomes through their progress and to identify their weaknesses and required support, educators can provide distinct learning processes with tailor-made feedback and support for each student to face any challenge and achieve learning objectives. The outcome is often enhanced learning and higher engagement levels of students [7,8].

In addition, LAT also enhances students' motivation by allowing the students to keep track of their learning progress and view firsthand the effects of their efforts, which would subsequently motivate their ongoing learning endeavors. It assists students in establishing their learning goals, overseeing their learning progress, and having a firsthand look at their fruitful efforts – which would further enhance their engagement and motivation towards learning [7]. Furthermore, LAT can clarify students' learning needs through enormous data concerning student behavior, learning patterns, and academic performance, data which is useful to understand students’ needs and develop learning experiences to meet such needs effectively [9].

In the perspective of teachers, it can enhance teaching effectiveness, equipping them with insights into the learning strengths, weaknesses, and styles of students, enabling them to develop instructions and design teaching strategies [10]. In fact, LATs use in education can provide several benefits to students, as evidenced by Karaoglan Yilmaz and Yilmaz [7], to teachers, as evidenced by Gutiérrez, Seipp [11], and to the whole learning institution as reported by Alzahrani, Tsai [12]. Some of the evidenced positive outcomes included enhancement of student learning outcomes, teaching effectiveness, student motivation, understanding of student needs, and institution decision-making. Through LATs, students can be supported in their successful endeavors, while the educational system is enhanced [1].

This is particularly important as higher learning institutions (HLIs) are always faced with the need to make decisions for program and operation management, including resource allocation, program evaluation and data-driven institutional strategy and policy decision-making. And without knowledge on the entailed factors and what they led to, such decisions may be difficult and challenging to reach. This may have been addressed through the use of LATs to support data-driven decision-making – tools that are able to establish student progress and achievement, management of resources and the like. In other words, HLIs can make use of LATs to reach informed decisions, enhanced students success and effectiveness of the learning programs while pushing the overall institution to eventual success. Such tools can be used by HLIs for effective management of programs and resources to achieve objectives and to meet students’ requirements in their learning process [3,13,14].

It is evident that the need to adopt LATs among HLIs is a must, given the fact that it can enhance student learning outcomes and their educational experiences. Nevertheless, this need has largely been ignored being that LAT implementation models are still lacking. This makes the institutions hesitate to implement and integrate LAT into their systems and processes, creating a hindrance to its extensive adoption among learning institutions. It is safe to say that institutions are wary of investing in something that does not present a clear guidance on its effective and successful implementation, use and benefits [3]. This issue is addressed in this paper through the development of a comprehensive and practical model for the adoption of LAT that institutions can use for effective system implementation, ensuring that HLIs can leverage its benefits and provide the students with the most enriching learning experience.

In clear terms, the study is an attempt to minimize the literature gap concerning the LAT adoption and practical usage in HLIs using the extended Technology Acceptance Model (TAM) in model development to ensure a thorough investigation into the determinants of such adoption. Towards this endeavor, the key determinants that drive the acceptance and implementation of LAT and their influence are examined along with the system's influence over the decision-making within institutions. The study findings are expected to provide practical insights to educational administrators and policymakers in their quest to include technology in the educational institutions.

Accordingly, there are three major research questions towards this research effort, the first of which is, “how can TAM be used for LAT adoption effectiveness in HLIs”? The second question is, “what are the critical factors influencing the acceptance and successful implementation of LAT in the institutions?” and the third question is, “how does the adoption of LAT influence the decision-making processes of HLIs?”. The above questions are directed towards the development of a practical model and framework upon which LAT integration in educational environment can be based.

Therefore, this paper is organized in the following way; the second part is dedicated to reviewing literature on LAT and the third one provides an outline of the model development. The fourth part of the paper describes the research methodology and the fifth part presents the discussion of results. This is followed by the sixth part, within which the contributions of the study are enumerated, and the seventh part which concludes the paper by presenting limitations and recommendations for future work, along with the summarized findings.

## 2 Related works on learning analytics tools (LATs)

In the field of education, the use of LAT has been garnering increasing attention in the past few years, particularly in its role in enhancing student learning outcomes and education system efficiency [1,12,[15], [16], [17], [18], [19]].

The purpose of this study is to determine several major factors influencing LATs adoption and use and to clarify their role in the same. Added to this, the research also aims to underline the importance of LATs in the education field for decision-making purposes. To begin with, several studies have been dedicated to the topic throughout the years, the more current ones including Ifenthaler, Gibson [20], who laid emphasis on the advantages of LATs use in education and in focusing on learning through the tools. They reviewed literature and conducted a Delphi study entailing the feedback of international experts for the identification of opportunities and challenges that comes with LAT use. Ifenthaler, Gibson [20]categorized the findings into four themes, namely development of data literacy, updated guiding principles and policies, ethical practices with data quality assurance, and lastly flexible user-centered design. The above themes were then developed into areas of action and strategy to be of assistance to policy-makers, researchers and practitioners to collaborate on maximizing the LATs effect on learners and instructors. Ifenthaler, Gibson [20]further stressed on data literacy, updated guiding principles, and continuous research to shed light on LATs effect on both learning and teaching processes.

In the same study line, Mutimukwe, Viberg [21] explored the higher education students' privacy concerns concerning learning analytics (LA) usage. They developed and employed the SPICE model, one which regards privacy concerns to be the core construct between perceived privacy risk, perceived privacy control, trusting beliefs and non-self-disclosure behaviors. Moreover, Mutimukwe, Viberg [21] gathered data from 132 students studying in three universities in Sweden via online survey and validated the SPICE model's ability to account for high variance in privacy concerns, trusting beliefs and non-self-disclosure behaviors. Based on their findings, perceived privacy risk is a strong predictor of privacy concerns and it affects non-self-disclosure behaviors. They also found that perceptions of privacy control and privacy risks are determinants of trusting beliefs. Mutimukwe, Viberg [21] recommended that LA system should be designed in line with the privacy concerns of students and that privacy-enhancing practices need to be established for system trust maintenance. Their study offered insights into the associations between student privacy concerns, trust and non-self-disclosure behaviors in light of LA in the higher education context.

Added to the above studies, Karaoglan Yilmaz and Yilmaz [7] examined the effects of personalized metacognitive feedback on the basis of learning analytics, on the online courses engagement of students. Their study adopted an experimental design and was carried out with the help of 68 first-year students, in Computing II course, in Turkey. The respondents were categorized into the experimental group (personalized feedback) and control group (no personalized feedback). In this regard, personalized feedback comprised of learning analytics reports and recommendations, with a student engagement scale used in pre- and post-test of the students. Based on the obtained results, the experimental group students obtained higher levels of engagement in comparison to their control group counterparts. Karaoglan Yilmaz and Yilmaz [7] reached to the conclusion that personalized metacognitive feedback built on LA is effective in enhancing online courses engagement of students. This means the integration of LA into online courses is important and so as the reception of personalized feedback in enhancing the engagement and learning outcomes of students.

Moving on to another LA type system, focusing on the Learning Analytics Dashboard (LADA), Gutiérrez, Seipp [11] examined its role in supporting decision-making process among academic advisers. The system was developed to fill the gap in educational support system and provide the required technical support with the accompanying descriptive statistics. LADA was employed and evaluated in two higher education institutions and based on the results, it offered benefits for experts and inexperienced advisers alike. In particular, the expert advisers were enabled to conduct evaluation of various scenarios for informed decisions in a similar time limit compared to traditional procedures, while inexperienced advisers could use LADA as a tool to reach decisions that are accurate and efficient. Such results were indicative of the potential extensive developments in both LA and academic advising.

Furthermore, LAT entails data analysis following data gathering from student interactions with educational and information technology to explain the learning process and enhance decision-making [22,23]. Although LAT has undergone many developments throughout the years, its implementation is still lacking in higher education. According to Gasevic, Tsai [24], an effective approach to LAT adoption in higher education calls for the consideration of the socio-technical nature as well as the innovation adoption complexities. They extensively reviewed literature and works concerning LAT in higher education, building from past business analytics adoption models and two projects, one in Australia and the other in Europe. They eventually put forward an approach that underlined the critical challenges that need resolution for a long-term successful LAT in learning and teaching. Such approach enables higher learning institutions to shift towards data-informed strategic decision-making, while supporting successful endeavors of students.

Aside from the above mentioned studies, other studies in literature have also supported the importance of LAT use in decision-making in the field of education, with the data-generated by the tools used for reaching informed decisions on allocation of resources, enrollment and retention of students, academic performance and effectiveness of various teaching strategies. Such use also promote evidence-based decision-making and assist institutions in determining the weak areas that need further support for successful outcomes [25,26].

It appears that there exists a dire need for more studies to be carried out on LAT considering a gap exists regarding its implementation and adoption. Such gap paves the way for future works to examine and explore the potential use of LAT in the education field, particularly in enhancing student outcomes and decision-making [14,27]. Hence, this study determines the level of LATs contribution towards HLIs decision-making process when it comes to student achievements, particularly in the face of various subjects and great number of students.

Hence, the unique contribution of this study pertains to LAT studies as it extends literature by developing a model for LAT implementation in HLIs, one that is notably lacking in past studies. Studies concerning this topic have emphasized mostly on the LAT's potential and the challenges encountered during its use, inadvertently disregarding the development of a structured approach for its adoption and use effectiveness. This study therefore used the extended TAM to minimize the literature gap and provided a clear and empirical framework for LAT integration into HLIs. This provides advantages for future practical applications setting the ground for future studies and stakeholders in the educational technology field.

## 3 Model development

Information technology adoption refers to the level to which an adopter looks forward to using technology for easy completion of tasks [28]. Another definition was provided by Proctor, Silmere [29], who described it as the adopter's initial intention and decision towards innovation use, while Rogers [30] indicated that it is the full use of creation as the most viable course of action to be made. More specifically, LATs adoption plays a key role in the delivery of education services, ensuring that the services implemented are effective, which will then contribute to their positive delivery, making adopters aware of the benefits of its use and complete implementation [12,21].

When it comes to technology on a global scale, majority of countries throughout the world has experienced the promotion of investing in technology innovation to generate enhancements for the whole educational system [31] and works dedicated to the education field evidenced that technology adoption remains ongoing [32], albeit LAT adoption is still rare.

This emphasizes the need to examine LAT innovation adoption among HLS in order so that they may be transformed into global educational leaders. Also, adoption of such innovation is a must in order to pave the way for the adoption of further innovative tools for learning enhancement.

In the next sub-section, the conceptual model and formulation of hypotheses are presented in detail.

### 3.1 Conceptual model of extended TAM

In the education sector, the investigation of LAT calls for using a model as a guide to the research process, as generally speaking, a model presents and establishes a systematic way to shed light and clarify the complex phenomenon of adoption and ensures the accurate, rigorous and replicable nature of the research [21,33].

In this regard, TAM is one of the most commonly used models used in literature for examining technology use and adoption in many fields, including education. It is a model that was initially developed as a theoretical framework to explain the factors influencing the acceptance and use of computer technology and since its inception it has been extensively adopted in many contexts with its reliability and validity established for the same purpose [34].

According to TAM, usefulness and ease of use are the main determinants of individual's intention towards technology use and in the case of LAT, the model is useful to understand the way teachers and students view usefulness and ease of use of LAT and the influence of both on their intentions towards adoption and use of the system within the teaching and learning realm [35].

In addition, the use of TAM as a model can determine the major influencing factors of LAT adoption and use in education upon which recommendations can be made to enhance the same, ensuring that LAT benefits are realized and that they effectively support successful student endeavors and efficiency of the whole education system [36].

**Fig. 1:**
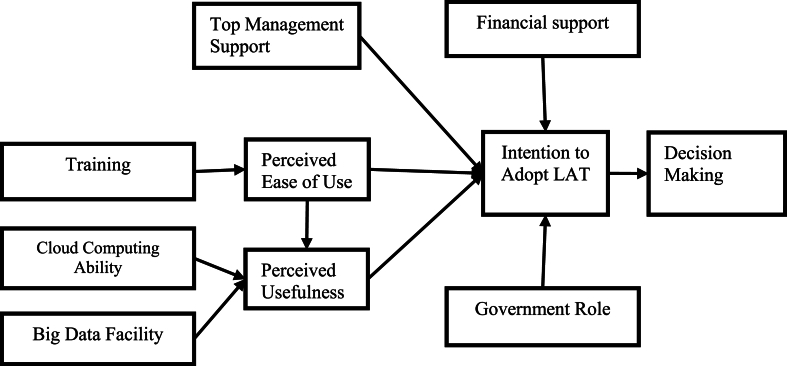
The conceptual framework for factors influencing the adoption of LAT

Lastly, TAM use is important when examining LAT in education as it presents a systematic and evidence-based approach towards understanding and explaining influencing factors on its adoption and use, making sure that the study is accurately focused, rigorous and can be replicated [27]. This study therefore aims to examine the influencing factors of LAT adoption through the behavioral intention of the students. The relationship of the factors and behavioral intention towards adopting IS has its basis on the studies that evidenced the effects of technological and organizational factors on intention to use (see Fig. 1). According to Davis's [37] TAM, both perceived ease of use (PEOU) and perceived usefulness (PU) influence behavioral intention towards IS and as such, on the basis of the literature reviewed, this study proposes the following conceptual LAT model.

According to Joyes, Gray [38], the successful implementation of an application hinges on its purpose and process of use and the proliferation of the same to potential users. In the education field, students are generally faced with issues in adopting applications owing to their lack of awareness of its objectives, purpose, structure and process flow [39] and thus, this calls for making sure that the system matches the structure of the campus so that the users will eventually realize its purpose and accept it [40]. In other words, the design of the system has to be distinct from the developer and administrator's whims and from the needs of the user. In relation to this, an ambiguous view of its use will have significant effects on its performance [41] and thus, implementation purpose and process need to be transparent so that the students can be motivated in their engagement and use of the system. Nevertheless, further studies are required to explore LAT in terms of its underlying processes to make sure that students are motivated and encouraged to use it and teachers use it in an effective and suitable manner. The relationship between process and outcome and the former's effects on the learner and his/her learning outcomes, also needs further examination [42].

### 3.2 Hypotheses formulation

In any given sector, technology use paves the way for enhancing the provided services quality and the personnel efficiency and effectiveness, thereby mitigating the expenses incurred by the organization [43]. Technology adoption is thus important for institutions as evidenced by several works on the subject [44]. Several positive effects report of technology adoption on organizations have been documented, albeit some studies also highlighted the barriers and challenges of IT adoption, and this holds true for the education sector. Thus, a look into the factors influencing technology adoption remains relevant in order to achieve technology adoption and implementation success. The following factors have been highlighted as technology adoption determinants in literature.

#### 3.2.1 Training

Studies in literature show that with adequate user's training on the use of systems, there is a higher likelihood of perceived ease of use (PEOU) and this means there will be a higher inclination to system adoption and effective use. Contrastingly, without training or lack of it, LAT may be difficult to use, leading to low PEOU level, and eventually ineffective technology use [[45], [46], [47]].

In a related study, Mukred, Yusof [48], explored the education staff experiences in regular use of technology and revealed that barriers to use included learning how to use the computer. In this regard, technology use can be improved by analyzing the user's computer skills, data entry and the overall system use, which need to be incorporated to the training sessions provided.

Additionally, training staff makes sure that risks do not crop up as these would lead to useless implementation and adoption, specifically in the case of LAT [49]. Lack of staff training will lead to lack of know-how on technology and eventually their lack of support for its use – this could mean low level of adoption [50]. Also, lack of training has also been evidenced to leading to discomfort among users when using the system (computers), and thus failed implementation [49,50].

In sum, both students and teachers need to fully know and be aware of the concept and practice of LAT when trained on its effective use. According to Händel, Wimmer [51], there are significant elements of training, which could ensure sufficient training and positive impact PEOU and in effect make them more inclined towards LAT adoption and thus, it is hypothesized that.

H1 Training has a significant relationship with perceived ease of use of LATs in HLIs.

#### 3.2.2 Cloud computing ability

The ability of the technology to store and access data and applications using remote servers is referred to as Cloud Computing Ability and in the case of LAT, it indicates the user's access to analytics data and tools from any place where internet connection exists, as opposed to just being confined to a distinct place [52].

Moreover, the strong Cloud Computing Ability of LAT would allow users to easily access analytics data and tools, thereby increasing their perceived usefulness of the system. Eventually, this will result in higher level of technology adoption and its effective use [53]. Weak Cloud Computing Ability of LAT contrastingly will lead to difficult access and use of analytics data, decreased perceived usefulness of the system and low level of adoption and use [54]. Hence, Cloud Computing Ability has a significant effect on the perceived usefulness of LAT and ultimately on its effective adoption and use.

Studies also supported the significant effect of Cloud Computing Ability on the adoption of LAT [53,[55], [56], [57]]. Thus, this study proposes the following hypothesis for testing.

H2 Cloud Computing Ability has a significant relationship with perceived usefulness of LAT in HLIs.

#### 3.2.3 Big data facility

This is the ability of technology to tackle huge data amounts in an efficient and effective manner. In LAT's case, this refers to its ability to gather, store and analyze large data amounts concerning the learners and their details [[52], [53], [54]].

Robust Big Data Facility of LAT can lead to easy access and analysis of large data amounts regarding learners, which could enhance its perceived usefulness and in effect promote higher levels of adoption and use. The contrary, which is weak Big Data Facility, may lead to difficulty to handle and analyze data, decreased perceived usefulness, and low adoption and use level among users [[52], [53], [54]].

Big Data Facility essentially has a significant effect on LATs perceived usefulness, and thereby its effective use and adoption. The perception that LAT is able to tackle large data amounts that would provide valuable insights would up the likelihood of its usefulness and adoption and thus, this study proposes that.

H3 Big Data Facility has a significant relationship with perceived usefulness of LATs in HLIs.

#### 3.2.4 Perceived ease of use

Perceived ease of use, based on Davis [58]TAM is the level to which an individual is convinced that using a specific system will be free of effort, and it is replicated in UTAUT as effort expectancy. The construct is perceived in this study from the viewpoint of management/employee of using LAT without or with minimal effort. This construct has been reported to influence intention towards adoption of technology and its use [59].

High perceived ease of use of LAT is expected to lead to the perception of a user-friendly and accessible technology and to its higher adoption and use, while low perceived ease of use of LAT will lead to difficulty in navigation and use of the system, low perceived usefulness and low adoption and use [60].

On the whole, perceived ease of use of LAT has a significant effect on its perceived usefulness and thereby, its adoption and use effectiveness. A more user-friendly technology that is accessible naturally leads to its higher perception of usefulness and higher adoption, while that which is not user-friendly could lead to low perception of usefulness and thus, lower rate of adoption. This study proposes that.

H4 Perceived ease of use of LAT has a significant relationship with its perceived usefulness in HLIs.

Related studies in literature [61] supported the influence of perceived ease of use on the academicians’ intention towards IT use. Additionally, adoption intention and actual use of IT was found to be affected by the perspectives and beliefs of users, encompassing both perceived ease of use and perceived usefulness that are both deemed to be the major antecedents of IS adoption [35].

Meanwhile, Arpaci [62] revealed that perceived ease of use also influenced the intention to use the system and Mosweu, Bwalya [63] supported such influence on both intention to use and system adoption and hence, this study proposes the following hypothesis for testing.

H5 Perceived ease of use has a significant positive influence on LAT adoption in HLIs.

#### 3.2.5 Perceived usefulness

This construct is described as the belief of the individual that system use would improve the performance on the job [58], and it is referred to as performance expectancy in UTAUT. In addition, perceived usefulness has a significant relationship with work productivity, money savings, time and motivation towards technology use in Yang, Wang [64] study.

In another related study, employing TAM to measure LATs acceptance among academic and professional employees, Ahmed and Ward [65] found that perceived usefulness of the system had a significant impact on intention to use. Also, performance expectancy and effort expectancy in UTAUT [66] are considered to be major antecedents of behavioral intention towards adopting IS.

Thus, this study describes perceived usefulness as the employees' perception of the usefulness of LATs when employed in achieving tasks and it is examined in relation to the system's ability to improve productivity, effectiveness and performance as adopted from past studies [63,67]. In this regard, the study proposes that.

H6 Perceived usefulness has a significant positive influence on LAT adoption in HLIs.

#### 3.2.6 Top management support

The top management's acknowledgement of the important contribution of technology function and content to the company's activities is referred to as top management support [68] and it has a positive or negative effect on the adoption of technology [69].

Other relevant studies explained that adoption of technology will fail if it lacks management and support from top management. Moreover, lack of top management support could lead to non-use of the system [68]. On the contrary, management support to using and adopting the system in the organization could lead to its eventual acceptance according to Wu and Gao [70]. They combined variables that prevent management support to look into the negative effects of users’ employment of the system and revealed that management support significantly affects adoption.

Studies also supported the positive effect of top management on IT adoption and acceptance, with two types of support documented, namely direct and indirect support. In the latter, vendors and consultants are used to promote adoption of the system, whereas in the former, IS staff plans such support from planning through development [71]. The studies also revealed that top management support positively affects the technology functions and performance.

Hence, support from top management has a significant influence over the intention of users and that of LATs adoption, whereby lack of such support could lead to adoption issues and those related to planning and developing the system and as such, this study proposes that:

H7 Top management support has a significant positive influence on LAT adoption in HLIs.

#### Financial Support

The LAT administration and implementation require sufficient and continuous funds and resources and similar to other management activities, programs and initiative management can only be sustained through funds assistance [72]. This entails long-term commitment from management and its awareness of the financial resources needed to be able to set aside sufficient budget for the program.

Additionally, financial support that covers monthly salary is a determinant of the commitment of the organization, specifically among developing nations' private universities personnel. This was revealed in the context of Nigeria by Popoola [73] in a study focusing on the socio-economic constructs, including monthly salary as determinants of management commitment among record management workers in private universities. On the basis of the obtained results, significant relationships existed among socio-economic factors and the commitment of the employees – among the factors, monthly salary had a significant relation with the respondents’ organizational commitment.

In the same caliber of study, Chen, Mou-Te Chang [74]highlighted the need for organizations to exert effort and financial resources for system activities management. In other words, LAT implementation needs appropriated resources and costs, with the inclusion of its initial establishment, implementation, maintenance and updates. Thus, adoption and implementation of the system can fail in the face of lack of funds and resources [75].

On the whole, technology adoption depends on financial support as evidenced by the positive influence that the latter has on adoption (e.g., LAT) and implementation, which would lead to enhanced future information efforts. The study looks into the influence of financial support on the adoption of LAT in the educational institutions, and therefore proposes that.

H8 Financial support has a significant positive influence on LAT adoption in HLIs.

#### 3.2.7 Government role

Government role is described as the role played by the government in promotion and support of technology adoption in specific circumstances [76]. In the context of this study, government support and incentives for the LATs adoption among HLIs can have a positive impact on the inclination of such institutions towards the adoption.

This may be exemplified by the provision of funding for developing and implementing LATs, provision of tax incentives towards technology adoption and development of regulations and standards for LATs use in the institutions [77,78]. These can contribute to the high perceived benefits and mitigation of perceived risks associated with LATs adoption, and eventually leads to higher level of technology adoption and its use effectiveness.

On the contrary, without support from the government for LATs adoption in HLIs, the institutions inclination towards its adoption may wane, in which case, perceived risks and perceived benefits of technology adoption will heighten and dip respectively – eventually lower adoption and ineffective technology use will ensue.

Hence, government has a key role in LATs adoption among HLIs whereby it can positively influence such adoption – the opposite of which, lack of government support can have an adverse effect on the technology adoption and use effectiveness.

In sum, the study model examines government role in affecting LATs intention towards adoption among HLIs in testing the following hypotheses.

H9 Government role has a significant positive influence on LAT adoption in HLIs.

#### 3.2.9 Intention to adopt LAT factors and decision making

According to past studies [79,80], intention or behavioral intention is the end-user's intention towards new technology adoption. Generally, intention is the level to which the user has pre-formulated plans to perform or refrain from performing a particular behavior in the future [81], while behavioral intention is the indication of the readiness of the individual towards performing a specific behavior. Behavioral intention is considered to be an immediate determinant of behavior [82]. Intention, in the present study, is described as the level to which users are inclined towards trying out or putting in effort to perform the behavior of technology adoption and use.

Moreover, behavioral intention towards technology is the driver behind actual behavior with three predicting factors (subjective norm, perceived behavioral control and attitude) [66]. Also, in their comparison of different technology acceptance models, Ahmed and Ward [83] aimed to examine portfolio acceptance behavior in terms of personal, academic and professional facets and found a positive direct effect of perceived ease of use on perceived usefulness and the same of perceived ease of use on intention.

In terms of decision-making, which is an evaluation process of available options and choices based on information, preferences and values, when it comes to LAT, the construct is the process of deciding whether to adopt the technology or refrain from such adoption. An individual with strong behavioral intention towards LAT adoption is more likely to engage in deciding to examine the pros and cons of its adoption. This may also lead to the higher chance of making a decision to adopt it through an informed and deliberate decision process. Contrastingly, an individual with low behavioral intention towards such adoption is not as likely to engage in deciding whether or not to adopt the technology [26].

Hence, behavioral intention towards technology adoption significantly influences the decision-making of users in LATs case. Moreover, a strong behavioral intention can result in informed and deliberate decision-making process and a higher chance of adopting LAT, whereas a weak behavioral intention can result in refraining from deliberating its adoption and thus lowers the chances of such adoption. Therefore, this study hypothesizes the following.

H10 Behavioral intention to adopt LAT has a significant influence on decision-making in HLIs.

**Fig. 2:**
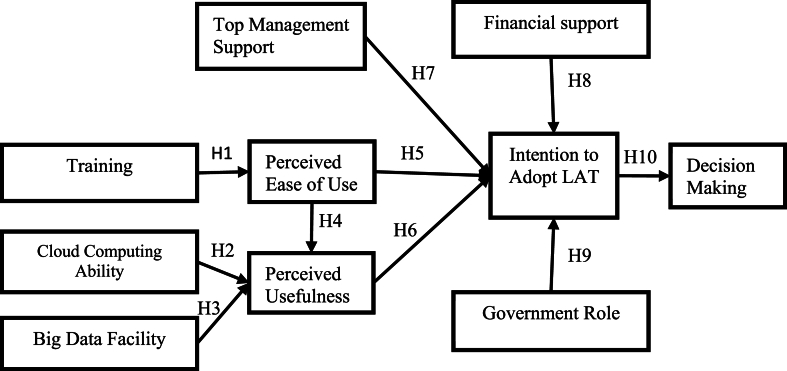
Conceptual framework with hypothesis.

Fig. 2 shows the proposed conceptual model for the LA adoption at HLIs to improve decision-making.

## 4 Methodology

Questionnaire tool was used as the main instrument to gather data and achieve the objectives in this quantitative study. The data gathered was exposed to quantitative analysis whereby the variables in the model were examined.

First, a sampling frame of employees was obtained from HLIs through the use of stratified sampling method on the basis of three position levels of the respondents. According to Creswell [84], this sampling method is suitable for the different population groups. The groups comprised of academicians, managerial employees and administrative employees. There were a total of 275 respondents used in the study.

The second step involved the use of Partial Least Squares-Structural Equation Modeling (PLS-SEM) to analyze data and the approach was chosen owing to its proficiency in addressing complex models that cover numerous constructs and relationships that played a role in the examination into the LAT adoption determinants [85,86]. Moreover, PLS-SEM was considered to be appropriate to be used for developing the theory and for addressing constructs, both reflective and formative, with the robustness of the approach accommodating smaller sized samples and data that is devoid of normal distribution [85]. The size of the sample is 275 respondents that occupy different positions in HLIs and thus further justifying the use of PLS-SEM. Accordingly, the method is used to conduct a comprehensive analysis of the variables’ interrelationships within the model, while making sure that the findings reliability and validity are established, in the face of the potential presence of multicollinearity and the non-normal distribution of data.

### 4.1 Questionnaire construction

The survey method was used to describe the phenomenon and examine the cause behind it (low level of LAT in HLIs). The survey essentially enables the researchers to gather data from the respondents and measure the variables effects as stated in the formulated hypotheses [87]. The questionnaire method coupled with the exploratory approach produces the best results. The questions used in this study are shown in Table 1.

**Table 1:** Questions on exogenous factors.

Factor	Adapted from
Perceived Ease of Use	[58]
Perceived Usefulness	[58]
Top Management Support	[88]
Financial support	[89,90]
Training	[91]
Government Role	[92,93]
Cloud Computing Ability	[94]
Big Data Facility	[52]
Intention to adopt LAT	[95]
Perceived Decision Making	[48,96]

The respondents’ gender, age, level of education, job experience in years and type of jobs were obtained in the last section of the questionnaire.

The conditions for inclusion in this study were developed to facilitate representation of sample of individuals who play a part or are influenced by LAT adoption in Higher Learning Institutions (HLIs). The sample units were chosen owing to their positions in HLIs being faculty members, administrators and IT staff who have a hand in decision-making processes when it comes to LAT. As for the conditions for exclusion, it is in contrast to that of inclusion, that is, individuals that held no position in the HLIs and no direct involvement with the LAT decision-making were excluded. The data collection was conducted by distributing questionnaire copies using the emails of the employees in Saudi public HLIs – such cross-sectional distribution ensured the achievement of a sample representative of different viewpoints and experiences. The distribution approach also made sure that the study findings include actual LAT adoption cases when it comes to its use and adoption in HLIs. This was further supported by the large sample size which enables generalization of conclusions concerning the determinants of LAT adoption in HLIs.

### 4.2 Reliability and validity

The reliability of results refers to the consistency and stability of the results characteristics and a study with valid and reliable results achieves an outcome that remains similar when replicated. In the case of SEM, Kline [97] explained that every variable requires a corresponding variable and valid measurement for consistent measurement. Thus, this study made use of Cronbach's Alpha coefficient to ensure reliability of the instrument. This method has been widely used in literature by majority of studies to establish internal consistencies and reliability of instruments. Accordingly, the instrument items were measured using Likert scales and an alpha value of 0.60 was considered as the acceptable level. Alpha value closer to 1 indicates higher reliability and internal consistency. This study found alpha values to vary from 0.825 to 0.875 as presented in Table 2, indicating good reliability levels.

**Table 2:** Initial reliability of instruments used.

Variable	No of Items	Cronbach's Alpha
Training	5	.875
Cloud Computing Ability	5	.862
Big data Facility	4	.825
Perceived Ease of Use	5	.837
Perceived Usefulness	5	.869
Top Management Support	5	.873
Financial Support	5	.875
Training	5	.862
Government Role	4	.825
Intention to Adopt	5	.856
Decision Making	5	.867

Relevant past studies on validation heuristics like Churchill Jr [98] and Brown, Suter [99] contended that items with alpha coefficient values lower than 0.60 are deemed unacceptable and should be dropped. Alpha coefficients of all 65 items in the questionnaire were exposed to reliability test.

With regards to the validity, Creswell [84] referred to it as the significance of the results and hence, it entails looking at the internal and external validities. Accordingly, Vargas-Hernandez et al. (2012) described internal validity of the instrument as the measure of its quality and the quality of the control variables, while MacKenzie, Podsakoff [100] described external validity as the results generalizability in particular cases.

Added to the above, testing validity establishes a bias-free data and an error-free instrument and validity test comes in different forms, including content validity, criterion validity and construct validity. Content validity is the scale of the measured characteristics properties which are obtained based on the personal judgment of the experts in the field [101], face validity is the exploratory type of content validity which are obtained based on item's cursory observation, logical validity is the evaluation of the intuitive items, and lastly criterion validity has predictive and concurrent validities as its two dimensions.

Aside from the above validity types, construct validity is the outcome of the construct measurement using two or more independent approaches, and this entails testing convergent, discriminant and nomological validities [102]. Scale validity is another type of validity which reflects the information and scale strength relayed through the achievement of the application used for testing [97].

## 5 Results

Under this section, the study findings and their interpretation are presented in detail.

### 5.1 Profile of demographic variables

This section is dedicated to presenting the demographic information of the respondents to clarify their characteristics and the relationship of the characteristics to the results and findings as seen in Table 3. In this regard, demographic information has proven its usefulness as a valuable tool in examining correlations and potential connections among the survey results. The survey respondents’ numbered 275 employees working in the government HLIs, from which demographic data constituting age, gender, education level, experience years and occupation type were obtained. The next sections detail the survey results analysis of such information.

**Table 3:** Summary of the personal characteristics of the respondents.

Variable	Types	Frequency	Percent	Valid Percent
Gender	Male	**197**	**71.64%**	**71.64%**
	Female	78	28.36%	28.36%
	**Total**	275	100%	100%
Age	Less than 25 years	5	1.82%	1.82%
	26–29 years	37	13.45%	13.45%
	30–39 years	85	30.91%	30.91%
	**40**–**50 years**	**130**	**47.27%**	**47.27%**
	Over 50 years	18	6.55%	6.55%
	**Total**	275	100%	100%
Qualification	Diploma	12	4.36%	4.36%
	**Bachelor**	**122**	**44.36%**	**44.36%**
	Master	120	43.64%	43.64%
	PhD	21	7.64%	7.64%
	Total	275	100%	100%
Job	Academician	**156**	**56.73%**	**56.73%**
	Managerial	76	27.64%	27.64%
	Administration	43	15.64%	15.64%
	Total	275	100%	100%
Experience	Less than 4 years	35	12.73%	12.73%
	5–7 years	70	25.45%	25.45%
	**8**–**10 years**	**88**	**32.00%**	**32.00%**
	10–15 years	39	14.18%	14.18%
	More than 15 years	43	15.64%	15.64%
	Total	275	100%	100%

Based on the results of the analyzed of the 275 government employees’ data, majority of the respondents (71.64%) were made up of male respondents, while the rest (28.36%) were female respondents. Added to this, majority of the respondents fell in the age range of 40–50 years of age (47.27%), then those in the age range of 25–20 years of age (27.2%), and finally, those in the age range of 30–40 years of age (24.8%). Most of the employees had bachelor degrees (44.36%0, some had master degrees (43.64%), others had PhD (7.64%) and the remaining (4.36%) had course diplomas.

According to the analysis of the demographic characteristics in light of the experience and type of occupation of the respondents, most of the respondents worked as academics (56.73%), some held managerial positions (27.64%) and others held administrative positions (15.64%). Finally, most of the respondents worked for 8–10 years (32%) while the least number of them worked for 4 years (12.73%). These quantitative findings reflect a clear picture of the respondents’ characteristics and experience.

### 5.2 Measurement model assessment

**Fig. 3:**
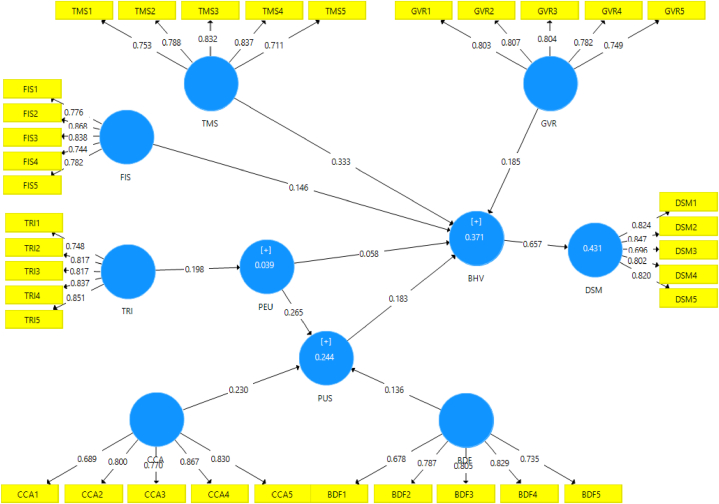
The Measurement Model of the Study (PLS algorithm results (regression weights)).

The measurement model construct reliability and validity reflect the measurement of the constructs while the assessment reflects its goodness-of-fit. The study used PLS algorithm to obtain the model's regression weights (see Fig. 3) on the basis of PLS 3.0.

#### 5.2.1 Model fit indicators-goodness-of-fit

Literature shows that goodness of fit in PLS-SEM, which is commonly used to test theories and confirm them, is missing [86]. Bentler and Huang [103] claimed that goodness-of-fit measures have been initiatives in PLS-SEM, while Henseler, Dijkstra [104]brought forward the standardized root mean square residual (SRMR) for measuring the squared discrepancy between the observed correlations and those implied for the model for its confirmation. To this end, a suitable match is deemed to have a value that is lower than 0.08, and in this case, the model fit was confirmed using PLS (SRMR = 0.07).

#### 5.2.2 Measurements of construct validity using confirmatory factor analysis

The way a set of items encapsulate the ideas they are developed to measure is known as their construct validity. The items used in this study were adopted from previous studies and have already been validated. The items and their corresponding loadings are tabulated in Table 4, confirming the recommended significant loadings on their corresponding constructs [105].

**Table 4:** Constructs, items, and confirmatory factor analysis results.

No	Variable	Code	Factor Loading	Cronbach Alpha	Composite Reliability	AVE
1	Training (TRI)	TRI1	0.748	0.873	0.908	0.664
2		TRI2	0.817			
3		TRI3	0.817			
4		TRI4	0.837			
5		TRI5	0.851			
6	Cloud Computing Ability (CCA)	CCA1	0.689	0.854	0.894	0.629
7		CCA2	0.800			
8		CCA3	0.770			
9		CCA4	0.867			
10		CCA5	0.830			
11	Big Data Facility (BDF)	BDF1	0.678	0.827	0.878	0.591
12		BDF2	0.787			
13		BDF3	0.805			
14		BDF4	0.829			
15		BDF5	0.735			
16	Perceived Ease of Use (PEU)	PEU1	0.767	0.846	0.889	0.615
17		PEU2	0.787			
18		PEU3	0.772			
19		PEU4	0.809			
20		PEU5	0.787			
21	Perceived Usefulness (PUS)	PUS1	0.758	0.818	0.873	0.579
22		PUS2	0.826			
23		PUS3	0.751			
24		PUS4	0.729			
25		PUS5	0.735			
26	Top Management Support (TMS)	TMS1	0.753	0.844	0.889	0.617
27		TMS2	0.788			
28		TMS3	0.832			
29		TMS4	0.837			
30		TMS5	0.711			
31	Financial support (FIS)	FIS1	0.776	0.862	0.900	0.645
32		FIS2	0.868			
33		FIS3	0.838			
34		FIS4	0.744			
35		FIS5	0.782			
36	Government Role (GVR)	GVR1	0.803	0.850	0.892	0.623
37		GVR2	0.807			
38		GVR3	0.804			
39		GVR4	0.782			
40		GVR5	0.749			
41	Behavioral Intention to Adopt LAT (BHV)	BHV1	0.787	0.825	0.878	0.589
42		BHV2	0.775			
43		BHV3	0.765			
44		BHV4	0.790			
45		BHV5	0.718			
46	Decision Making (DSM)	DSM1	0.824	0.858	0.898	0.639
47		DSM2	0.847			
48		DSM3	0.696			
49		DSM4	0.802			
50		DSM5	0.820			

Acceptable composite reliability is higher than 0.70 and the values found in this study ranged from 0.873 to 0.908. Cronbach's alpha values were also acceptable as they varied from 0.818 to 0.873. Moreover, the values of AVE all exceeded 0.50, and ranged from 0.579 to 0.664. The CFA results are presented in Table 4.

### 5.3 Structural model analysis

**Fig. 4:**
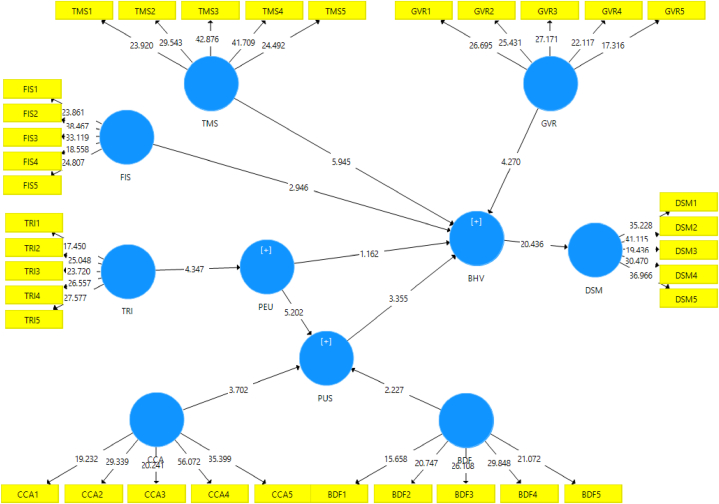
The Structural Model of the Study (PLS bootstrapping (T Statistics)).

The formulated hypotheses propose the relationships among the constructs and this phase of the analysis tests such relationships. Accordingly, this study made use of Smart PLS 3.0, and the path coefficient findings are displayed and presented in Table 5 and Fig. 4.

**Table 5:** Hypotheses testing results.

Hypothesis		β	T-value	*P*-Value	Result	R^2^
	Behavioral Intention to Adopt LAT					0.371
H1	Training → Perceived Ease of Use	0.198	4.347	0.000	Supported	
H2	Cloud Computing Ability → Perceived Usefulness	0.230	3.702	0.000	Supported	
H3	Big Data Facility → Perceived Usefulness	0.136	2.227	0.027	Supported	
H4	Perceived Ease of Use → Perceived Usefulness	0.265	5.202	0.000	Supported	
H5	Perceived Ease of Use → Intention to Adopt LAT	0.058	1.162	0.241	*Not Supported*	
H6	Perceived Usefulness → Intention to Adopt LAT	0.183	3.355	0.001	Supported	
H7	Top management support → Intention to Adopt LAT	0.333	5.945	0.000	Supported	
H8	Financial support → Intention to Adopt LAT	0.146	2.946	0.004	Supported	
H9	Government Role → Intention to Adopt LAT	0.185	4.270	0.000	Supported	
	Decision Making					0.431
H10	Intention to Adopt LAT → Performance	0.657	20.436	0.000	Supported	

The statistical analysis results that the data was exposed to showed general support for the entire formulated hypotheses, with the exception of H5, which proposed the relationship between perceived ease of use and perceived usefulness (β = 0.058, t = 1.162, p 0.241).

The examination of the strength of the influence of the external latent construct on the endogenous latent construct is normally carried out by calculating the impact size (f^2^) [106] and this is done through the relative change in the (R^2^) value [107]. On the basis of Cohen's [108] rule of thumb, (f^2^) value of 0.35 is deemed to be big, that of 0.15 is medium and that of 0.02 is small. Table 6 displays the (f^2^) values obtained in this study.

**Table 6:** Effect size f.^2^.

	BHV	DSM	PEU	PUS
BDF				0.017
BHV		0.758		
CCA				0.045
FIS	0.03			
GVR	0.05			
PEU	0.004			0.079
PUS	0.039			
TMS	0.126			
TRI			0.041	

Moving on other model fit indicators, Variance Inflation Factor (VIF) and tolerance values both measure the multicollinearity level and can be used independent of each other or in combination [109]. Specifically, the tolerance values indicate the amount of unaccounted-for variation in one indicator of a specific construct that are not accounted for by various indicators in the same block, whereas the VIF measures collinearity being that it is the inverse of tolerance values [86].

Concern exists if the highest VIF value exceeds 10 [86], but others claim that it should not exceed 5. Also, tolerance value should not exceed 0.1, while 0.2 is indicative of a potential issue [86]. However, in this study, no sight of significant multicollinearity was found among the predictor variables (see result in Table 7), as all VIF values remained lower than 5, which means the predictor variables contributed to the variance in the dependent variable in a way that such contributions did not overlap.

**Table 7:** Multicollinearity test via variance inflation factor (VIF).

	BHV	DSM	PEU	PUS
BDF				1.482
BHV		1		
CCA				1.552
FIS	1.143			
GVR	1.081			
PEU	1.274			1.174
PUS	1.344			
TMS	1.408			
TRI			1	

The correlations among the constructs should be lower than the squared of the AVE and that the latter needs to be higher than 0.50. The squared AVE values in this study were all found to exceed the correlations among the constructs, confirming sufficient discriminant validity as seen in Table 8.

**Table 8:** Discriminant validity.

	BDF	BHV	CCA	DSM	FIS	GVR	PEU	PUS	TMS	TRI
BDF	**0.769**									
BHV	0.441	**0.768**								
CCA	0.560	0.454	**0.793**							
DSM	0.400	0.657	0.385	**0.800**						
FIS	0.317	0.333	0.314	0.326	**0.803**					
GVR	0.346	0.315	0.339	0.341	0.178	**0.789**				
PEU	0.304	0.312	0.365	0.268	0.151	0.205	**0.784**			
PUS	0.346	0.412	0.404	0.376	0.195	0.187	0.391	**0.761**		
TMS	0.390	0.514	0.425	0.433	0.329	0.174	0.370	0.432	**0.786**	
TRI	0.229	0.315	0.276	0.282	0.502	0.149	0.198	0.135	0.456	**0.815**

## 6 Discussion and interpretations of the study

With the advancement of technology, there are more opportunities to enhance the learning and teaching experience for students and instructors and provide more personalized education [2,5]. LATs is one such technology whose use has been found to be beneficial in the field of education, particularly in higher education [4,[110], [111], [112]]. This research primarily aimed to develop and propose a LAT adoption model for HLIs that contains the factors that influence LAT adoption within the institutions. The study extends literature concerning adoption of LAT through an extended TAM model. Several variables were analyzed with their relationship to adoption including training, big data facility, cloud computing ability, perceived ease of use, perceived usefulness, top management support, financial support, government role, behavioral intention towards LAT adoption, and institutional decision-making. Notably, LAT technology has shown rapid and dynamic development owing to the significant investments to its launching and usage.

The findings provide insights into the adoption factors affecting behavioral intention towards LAT adoption among HLIs that improves the ability of the institutions to make informed decisions. The findings also supported the significant direct effects of top management support, financial support and government role on LATs adoption, with both big data facility and cloud computing ability revealed to be important in forming perceived usefulness of the system. The findings further confirmed the significant contribution of training on perceived ease of use of LATs and in turn, its adoption's role in enhanced HLIs decision-making.

Ease of use appears to have a significant role in technology adoption models but in case of LAT adoption in HLIs, its influence is lower in strength compared to other factors contributing to the effectiveness of LAT, the consistency of the tool with the goals of the institution, and its contribution to enhancing the operational outcomes of the educational institutions [14,20]. LAT adoption is largely dependent on the various academicians, managerial staff and administration's priorities as opposed to just their perception of the ease of use of the tool. More specifically, academicians are more concerned with LATs effectiveness and educational impact and its potential to improve teaching and research processes, while managers are concerned with LATs alignment with the objectives of the institutions and the benefits the institutions can reap from it in the long run (e.g., data-driven decision-making). On the other hand, administrators in HLIs are more concerned with LAT's integration with the current systems, the requirements for training and its operational efficiency rather than the system's ease of use. These different priorities to adopting LAT represent the various requirements and objectives of the different HLIs personnel, which could be the reason behind the lower influence level of ease of use over technology adoption.

Additionally, the study makes various implications to theory, beginning with the development of the LAT adoption model for HLIs for enhanced decision-making. LAT adoption models are still needed in literature, specifically those in the field of education (learning and teaching). An extensive picture of LAT use, its capability, effectiveness and efficiency in the institutions were provided by the findings through empirical testing of the variables influence over adoption.

As for the study's practical implications, it has several ones for decisions makers running HLIs and these lie in the understanding of the major influencing factors of LAT adoption, through which informed decisions can be reached concerning the technology investments and the adoption strategies. In relation to this, HLIs authorities can make use of the findings in their practical daily activities.

Added to this, the findings also clarify the role and influence of the factors for researchers and practitioners concerned with the technology adoption area in learning institutions. The study adopted specific techniques, formulated hypotheses and applied the most suitable measuring tools and offered a detailed explanation of the design and structure of the research. Through the integration of data obtained through the survey, the study made sure that the results obtained were accurate. The investigative tools selection was also supported throughout the research, enabling the study to contribute to the SEM literature in technology adoption area considering the model was used for structural model testing.

Unfortunately, literature is still lacking when it comes to LAT and its function in promoting educational institutions productivity and as such, this study minimizes the literature gap in this matter, particularly in Saudi Arabia and other developing nations [1,3,27,113]. Also, owing to the lack of standardized information/guidelines on LAT implementation, the study fills the gap and can be used as guideline for future endeavors in this area. Evidently, the study has ample benefits for educational administrations and LAT developers who can use the findings to enhance the proper adoption and use of the system, and ensure that system design and features of the system are capable of meeting user expectations respectively. Also, Big Data Facilities administrators in the education field can leverage the findings to make a proper plan and strategy for LAT adoption success.

Through the illustration of the significant influence of factors, namely training, big data facility, cloud computing ability, and organizational support on the adoption and use of LAT, this research provides insight into the entailed complexities when implementing LAT in HLIs. Such insight is core to the development and accurate refining of models dedicated to implementing LAT in an approach that covers multiple dimensions (technological, administrative and policy) for integration success. The underpinning model, extended TAM, stresses on perceived usefulness and ease of use in their influence over the adoption, which supports the need for user-centered design of LAT. Our study explored the way the factors have direct influence over the HLIs decision-making process, highlighting the need for effective implementation in a way that is supported by empirical findings. The study also evidenced the ability of the proposed LAT adoption framework in tackling the literature gap and paves the way for future studies to develop the accuracy and effectiveness of the LAT adoption frameworks. The minimizing of the gap between theoretical models and practical adoption contributes to both theory and practice via a basis upon which educational institutions can build upon when using LAT for enhanced results.

Another important aspect of this research is its venture into the interrelationships between technology and pedagogy and its emphasis on the important role that LAT plays in facilitating unique educational experience. The study highlights the need for continuous teamwork between developers, educators and policymakers in a way that ensures the technical advancement of LAT and its pedagogical relevance and alignment with the objectives of educational institutions. The study provides evidence of support to the critical need for ongoing feedback and evaluation mechanisms when it comes to LAT, particularly on the way real-time data can be used for adapting teaching curricula and techniques towards the current needs of the students. With regards to global education, the findings furnish information on the adoption and integration of LATs in educational settings, regardless of culture and educational institution type. The implications of the study are not confined to higher education surroundings but also other educational sectors, essentially addressing the extensive discourse on technology-enhanced learning. The research not only minimizes the gap in literature but also paves the way for future works to leverage the findings and ultimately lead to developing educational practices that are effective, innovative, inclusive and worldwide.

The study findings support those of past studies, which found the significant role of perceived usefulness and management support in LATs adoption within institutions of higher learning. Aligned with [114] study, which is concerned with the perceived benefits and their effect on the inclination of the educators and administrators in adopting new technologies, this study reinforces the importance of perceived usefulness in LAT adoption success. As for management support, in Alzahrani, Tsai [115] study, the authors found top management support to be crucial for technology implementation success in educational context. This alignment manifests the consistent result of the examined factors role in LAT adoption and in turn, the effect on the decision-making process of HLIs.

However, in contrast to some past studies, this study revealed that perceived ease of use had no significant effect on intention towards LATs adoption, indicating a deviation from the reported findings. This study also found ease of use to have lower effect on LAT implementation compared to other factors such as technological infrastructure, perceived usefulness and institutional support – a result which may be attributed to the dynamic technological use of educational professionals which shifts the direction towards strategic and long-term benefits that can be reaped from the tools from their user friendly characteristic.

This study found technological factors namely, big data facilities and cloud computing as main determinants of perceived usefulness and this coincides with the current trends in literature. Along a similar line, studies [52,116] emphasized the increasing importance of advanced technological infrastructure in improving the educational tools perceived value and effectiveness. Also, the examination of the government's role in LAT adoption opens the doorway to another under-studied aspect in literature, contributing to a new dimension to the adoption discourse, and implying that external policy and regulatory environments with internal institutional dynamics, do play a role in LATs adoption process among HLIs.

## 7 Conclusion

In the higher learning institutions, there is a notable role for LATs to play in improving decision-making and this involves the provision of data-driven information into the learning and engagement of students. LATs are directly related to the formation of informed educational initiatives, providing benefits such as, enhanced learning development, personalized learning experiences, and enhanced student engagement, which ultimately leads to higher learning retention. The study is an attempt to mitigate the gap in literature concerning LAT adoption factors and their effect on HLIs decision-making process. The study used TAM as the underpinning theory to examine the determinants of LAT adoption and based on the findings, big data facility, cloud computing, perceived usefulness and training have significant roles to play in LAT adoption success. Other determinants from the findings include perceived usefulness, government, top management and financial support – all of which contribute to intention towards LATs adoption, and in subsequence, on the decision-making of HLIs. Therefore, decision-makers in HLIs can make use of the findings while researchers can build on them to delve deeper into big data and digital transformation and their facilitating of evidence-based decision-making optimization in today's digital realm.

### 7.1 Contributions

Moving on to the study contributions, the study's primary contribution is to assist HLIs in enhancing their performance and productivity through quality LAT content management, specifically in the developing nations, like Saudi Arabia. The study minimizes the literature gap and practice through the emphasis on LAT effective adoption.

In addition, the study contributes to both theory and practice – to the former by developing and proposing the LAT adoption framework for enhancing HLIs decision-making and by clarifying the factors that influence LAT adoption in the educational field. Literature is extended through the examination of the LAT functions in support of HLI performance and productivity through informed decisions.

The application of this study of extended TAM as the underpinning theory contributes to both theory and practice in LAT adoption among HLIs. In theory, the study extends TAM to educational technology rather than confining it to the traditional use in business and IT fields. This approach enriches the effectiveness of the model by stressing on sector-specific variables, particularly on those that determine LAT adoption success, while in practice, the research provides direction for the adoption and implementation of LAT in HLIs through the proposed structured framework. Such framework contains the relevant factors for LATs adoption success and subsequently informed decision-making in HLIs. Such contributions minimize literature gaps about educational technology and sets a precedent upon which future studies and practical applications can build upon in the quest for LAT deployment effectiveness in HLIs.

Practical contributions also include the usefulness of the study as a guide for institutions towards proper and effective LAT adoption on the basis of the adoption determinants and drivers. Data obtained was based on users' perceptions and intentions towards LAT adoption and the role of the system in enhancing HLIs employees' performance and decision-making. The findings are useful for the education authorities, government policymakers, and academic institutions in strategies and policies development. The study's provided empirical and practical information concerning the effective adoption of LAT is invaluable for the education sector's optimum performance.

### 7.2 Limitations and suggestions for future studies

The study's focus is directed towards HLIs and their adoption of LAT, and the impact of such adoption on their decision-making process. Notably, the findings may not apply to the private sector institutions as there are differences in adoption issues between the public and private entities. Further studies are thus needed to examine the generalizability of the framework and its applicability to the private institutions. In this regard, future studies can conduct testing on the applicability of the framework with its original factors and additional factors (e.g., culture).

As for the study limitations, the study solely focused on developing the LAT in institutions for employees and not for any other individual groups (e.g., students). To this end, future studies need to expand the examination by testing the applicability of the framework to individual and institutional performance. Extension of the findings is possible through an in-depth investigation into the relationships between the determinants of LAT adoption and the institutions performance.

The sample of the study was selected from the HLI institutions population, which is reflective of a single type of educational model, indicating that the findings in other educational models and institutions may differ. This limitation may be addressed by future studies examination of other institutional types and levels in their LAT adoption examination. Notably, the focus of the study is limited to several factors in light of their effects on intention towards LAT adoption among HLI employees. There are other factors left to examine that could have a potential role in driving or preventing intention to adopt. Future studies can incorporate the factors and test their influence on the attitudes and intentions of the users towards LAT adoption.

In the long-run, future studies dedicated to LAT implementation in HLIs can benefit from the extended scope and methodology of the study. They can cover various contexts, nation and institution-wise, and move further beyond specific regions to cover a more extensive global ground. Such investigation into a more extensive context will furnish enriching information of cultural and contextual effects on the adoption of LATs and similar technologies. At the same time, obtaining students' perspectives can provide a holistic perspective of the effect of LAT in HLIs. As the main users of LATs, students’ feedback can be invaluable for the enhancement of the tools effectiveness. Added to this, future studies can adopt a mixed-method approach (quantitative and qualitative methods) to delve deeper into the LAT adoption dynamics and clarify the human interactions with the technologies in terms of complexities and depth. Further longitudinal studies may also be used to assess the effect and long-term effectiveness in adopting LAT, and in turn, its effect on educational outcomes in the long-run. Lastly, emerging technologies like artificial intelligence and machine learning can be examined in light of their enhancement of LATs effectiveness and adaptability in a way that would contribute to their evolutionary development and sustain their invaluable contribution to an innovative educational process.

Finally, the study's focus targets the direct relationships when it comes to LATs adoption in HLIs, paving the way for studies to examine the indirect relationships. Such a step could delve deeper into the nuances of the adoption dynamics – for instance, the mediating role of organizational culture on the relationship between management support and LAT adoption, or the mediating role of user satisfaction on the relationship between perceived usefulness and long-term adoption. Such complex and indirect interrelationships would enrich knowledge and information regarding LAT adoption, which would consequently result in effective educational strategies.

## Ethics

This research has been conducted in accordance with the ethical principles outlined by the Sunway University Research Ethics Committee (SUREC). The study received approval under the reference number: SUREC 2023/075.

Additionally, the informed consent was obtained from all participants involved in the study. Participants were provided with comprehensive information regarding the nature, purpose, and potential risks and benefits of their involvement. They were assured of the confidentiality of their data and had the opportunity to ask questions before providing written consent.

## Funding

This research was supported by the Researchers Supporting Project number (RSP2024R244), 10.13039/501100002383King Saud University, Riyadh, Saudi Arabia and by the 10.13039/501100004515Universiti Kebangsaan Malaysia GUP 2022 061.

## Data availability

Data generated and utilized for analyses of results presented in this manuscript are available from the corresponding author on reasonable requests.

## CRediT authorship contribution statement

**Muaadh Mukred:** Writing – review & editing, Writing – original draft, Visualization, Validation, Supervision, Software, Resources, Project administration, Methodology, Investigation, Funding acquisition, Formal analysis, Data curation, Conceptualization. **Umi Asma’ Mokhtar:** Resources, Project administration, Funding acquisition, Data curation. **Burkan Hawash:** Software, Resources, Conceptualization. **Hussain AlSalman:** Software, Resources, Project administration, Formal analysis, Conceptualization. **Muhammad Zohaib:** Software, Resources, Project administration, Investigation, Formal analysis.

## Declaration of competing interest

The authors declare that they have no known competing financial interests or personal relationships that could have appeared to influence the work reported in this paper.
